# Early Marriages

**Published:** 1885-10

**Authors:** 


### Early Marriages.

By which we mean, under twenty-three for the woman and under twenty-eight for the man, are the misfortune and calamity of those who contract them. The constitution of the woman is prematurely taxed by early child-bearing, and is broken down before she is thirty-five, the age in which she ought to be in all the glory of matronly beauty, of social and domestic influence and power and enjoyment. But instead of this, in what condition does "thirty-five" find the great majority of American women? thin, pale, wasted, hollow cheeks, sunken and dark circled eyes, no strength, no power of endurance, with a complication of peculiar ailments, which, while they baffle medical skill, irritate the body and leave the mind habitually fretful and complaining, or what is less endurable, throw it into a state of hopeless passivity, of wearisome and destructive indifference to family, children, household, everything!

The influence which these things have on the manly ambition of the husband, is disastrous ; his solicitude and sympathy for his suffering wife, waste the mental power which ought to have been put forth in his business ; his time is diverted, whilst the reckless waste of servants unlooked after, and that unavoidable wreck and ruin to house and furniture and clothing, which is an inseperable attendant on every wifeless family ; these things, we say, soon begin to have a depressing effect on the energies of the young father and husband, who is but too often driven into do-nothing-indulgence, into reckless shifts, or into the forgetfulness of habitual drunkenness. All this time, the children are increasing in number, are more and more neglected, growing up in ignorance and idleness ; or if learning at all, having the more leisure to learn but too well, the habits and practices of ignorant, trifling, deceiving, blarneying, treacherous servants, for such the mass of them are, as we know by sorrowful experience, in all the large cities of this country.

A woman who begins to have children at eighteen cannot have that vigor of body and mind which is essential to a well-regulated household ; we say, therefore, to every young man,

Do not marry under twenty-eight for yourself, nor under twenty-three for your wife ; and remember, too, that the best dower a woman can bring you, is a sound constitution ; it is worth more to you than "*a fortune*," while its moral and physical effect on the future health and happiness of the children who may be born to you, cannot be measured by any array of dollars.

